# An analytic framework for conceptualisations of disease: nine structuring questions and how some conceptualisations of Alzheimer’s disease can lead to ‘diseasisation’

**DOI:** 10.1007/s11019-020-09963-2

**Published:** 2020-08-08

**Authors:** Kristin Zeiler

**Affiliations:** Department of Thematic Studies: Technology and Social Change, Linköping University, 581 83 Linköping, Sweden

**Keywords:** Alzheimer’s disease, Concept, Performativity, Diagnosis, Pathology, Disease, Diseasisation

## Abstract

According to the US National Institute on Aging and the Alzheimer’s Association (NIA-AA), Alzheimer’s disease (AD) should be understood as a biological construct. It can be diagnosed based on AD-characteristic biomarkers only, even if AD biomarkers can be present many years before a person experiences any symptoms of AD. The NIA-AA’s conceptualisation of AD radically challenges past AD conceptualisations. This article offers an *analytic framework for the clarification and analysis of meanings and effects of conceptualisations of diseases such as that of AD*. This framework consists of nine questions that allows us to determine how the conceptualisations of diseases, such as that of AD, link or decouple the following terms to/from each other: screening, diagnosis, pathology, disease (along the lines of what have been labelled as “biological-physiological” or “normative” conceptions of disease in philosophy of medicine), symptoms, and illness. It also includes questions regarding how specific decouplings open up for new categories through which people can understand themselves in new ways, and what spaces of possibilities specific conceptualisations (and their decouplings and linkages) open to. The article shows how specific decouplings/linkages can open up not only for the phenomena of pathologisation but also for a distinct, but related phenomenon here termed as diseasisation.

## Introduction

Dementia has been described as one of the major challenges facing health care systems (cf. the UK Department of Health 2012). Calls have been made for the early testing for dementia and interventions to increase dementia diagnosis have been carried out (see for example Burn et al. 2019; Krohne et al. 2011). Alzheimer’s disease (hereafter referred to as AD) dementia has been estimated to account for 70% of all cases of dementia (Kamentani and Hasegawa 2018), and much socio-political and scientific work specifically target AD.

New conceptualisations and diagnostic criteria for AD have been recommended (Dubois et al. 2007, 2010, 2014, 2016; Cummings et al. 2013; Jack et al. 2011, 2018; Sperling et al. 2011; McKhann et al. 2011) that challenge past conceptions. A workgroup convened by the US National Institute on Aging and the Alzheimer’s Association (NIA-AA), for example, has argued that AD should be understood as a “biological construct”, and that the diagnosis of AD be based on AD-characteristic biomarkers, irrespective of whether clinical symptoms are present (Jack et al. 2018, p. 536). This would allow a diagnosis of AD several years before a person has any symptoms. Concerns have been raised as to whether such an early diagnosis is more beneficial than harmful for the individual and ethically desirable (Schermer and Richard 2019; see also Karlawish 2011). There is currently no cure for AD. While social interventions can support individuals and people close to the patient, the drugs available primarily target symptoms, and result in temporary cessation or slowing of the rate of cognitive decline.

The aim of this article is to present an *analytic framework for the clarification and analysis of meanings and effects of conceptualisations of diseases such as that of AD*.^1^ This framework allows us to determine how the conceptualisations of diseases, such as that of AD, link or decouple the following terms to/from each other: screening, diagnosis, pathology, disease (along the lines of what have been labelled as “biological-physiological” or “normative” conceptions of disease in philosophy of medicine), symptoms, and illness. In this regard, I take inspiration from Björn Hofmann’s (2018, 2019) illuminating discussions of how the concepts of diagnosis, pathology, disease, harm, and suffering can be linked to or decoupled from each other, and I hope that the framework can contribute to the analysis of “shifts and continuities,” as put by Marianne Boenink, in conceptualisations of disease (Boenink 2016, p. 66).

Further, the framework draws on and takes inspiration from the ideas that “conceptual worlds are critical; they bring some observations, interpretative abilities, and options into focus while obscuring others” (Holstein 2000, p. 158), that specific conceptualisations of AD are parts of tangled conceptual webs of interrelated terms, and that new conceptualisations and categories can help to create new “spaces of possibilities” and make possible “new ways for people to be” (Hacking 1985, p. 165). For this reason, the framework also includes questions regarding the conditions under which the decouplings/linkages emerge; whether and if so how people can take up new categories, understand themselves in new ways through them and, when this is the case, whether and if so how these processes can help to shape what actions become (more or less) possible for them. I concur with the view of among others Boenink (2018, p. 216) that guidelines with disease conceptualisations and recommendations can have “*world making effects*” (italics in the original).

I use the analytic framework in order to examine the conceptual world of two recent AD conceptualisations: the conceptualisations suggested by the NIA-AA (Jack et al. 2011, 2018; Sperling et al. 2011; McKhann et al. 2011; Jack et al. 2018) and the one proposed by the International Working Group for New Research Criteria for the Diagnosis of AD (the IWG, see Dubois et al. 2007, 2010, 2014, 2016). By so doing, I hope to show how the analytic framework can enable conceptual clarification including clarification of implications of specific conceptualisations, and discussions of meanings and effects of AD conceptualisations.

The IWG recommendations have been developed by an international group of clinicians and researchers, many of whom convened at the initiative of Profs. Bruno Dubois, Philip Scheltens, and Howard Feldman in 2005. The term “IWG” is the term this group uses when referring to its recommendations (Dubois et al. 2014). The National Institute on Aging and the Alzheimer’s Association (NIA-AA) convened a workgroup to elaborate diagnostic guidelines for AD and update NINCDS-ADRDA guidelines, i.e. the guidelines from the US National Institute of Neurological and Communicative Disorders and Stroke (NINCDS) and the Alzheimer’s Disease and Related Disorders Association (ADRDA) (Jack et al. 2011), a few years after the IWG, in a US context. The NIA is a separate institute at the National Institutes of Health, and was created following recommendations made by a White House Conference in 1974. The NIA describes itself as “the primary Federal agency supporting and conducting Alzheimer's disease research” (https://www.nia.nih.gov/about). The Alzheimer’s Association (AA) is a US health organisation whose activities include organising fundraising events for Alzheimer’s research and support groups (https://www.alz.org/about).

In contrast with older conceptions of AD, those proposed by the IWG and the NIA-AA suggest that AD be understood as a continuum (Dubois et al. 2010; Jack et al. 2018; Dubois 2018). Further, the frameworks proposed by the IWG and the NIA-AA have both been designed primarily for the purpose of diagnosing individuals who are taking part in biomedical research. Such a framework would then help to “provide a common language to advance the scientific understanding of the preclinical stages of AD and a foundation for the evaluation of preclinical AD treatments”, as stated the NIA-AA in 2011 (Sperling et al. 2011, p. 281). While the NIA-AA’s early recommendations from 2011 also set out clinical criteria of recommendations for the symptomatic stages of AD (Jack et al. 2011, p. 260; see also Jack et al. 2018, p. 537), later recommendations from 2018 underscore that the framework is for research. The IWG at times refers to their framework as a diagnostic framework and states, in 2013, that it aims to integrate “molecular biomarkers of Alzheimer pathology into a diagnostic framework for earlier clinical diagnosis” (Cummings et al. 2013, p. 267). The idea of a neat confinement to research projects has been criticised. McCleery et al. stated when describing the NIA-AA recommendations that: “these proposals will inevitably shift the disease boundary in the clinic” and contribute a “powerful impetus to the spread of biomarkers into diagnostic practice” (2019, p. 176). Further, despite their similarities, the conceptualisations of AD made by the IWG and the NIA-AA differ significantly from each other, as this article will discuss.

The article agrees (to a certain extent) with the suggestion of Schermer and Richard (2019) that the IWG exemplifies older normative conceptions of disease (such as that of Nordenfelt 1987), while the NIA-AA exemplifies biological-physiological conceptions (such as that of Boorse 1977, 2014). However, it also engages with more recent NIA-AA recommendations, and argues that the conceptions proposed by both the NIA-AA and the IWG do not qualify as clear-cut examples of biological-physiological or normative conceptions of disease. Further, previous work has discussed the goals of AD conceptualisations, and their effects on the individual such as whether the diagnosis of preclinical AD will help to transform healthy individuals into patients; whether such a diagnosis is in people’s “best interests”, and whether it can change people’s self-perception. Societal effects discussed in previous work include a “medicalization of aging” (Schermer and Richard 2019, p. 144; Karlawish 2011). Concerns have been voiced about how a diagnosis of preclinical AD will be communicated to patients, and how to ensure that people diagnosed with preclinical AD will not be discriminated (Karlawish 2011). This article adds a focus on how AD conceptualisations mobilise specific conceptual decouplings/links, and how these decouplings/links can strengthen a focus on biomarkers and/or clinical symptoms. It discusses the implications of some conceptual decouplings/links for individuals, for society, and for clinical practice, and suggests that the NIA-AA’s conceptualisation of AD can encourage a phenomenon termed as diseasisation—that is distinct from yet related to pathologization. Finally, while I discuss AD, I hope that the framework also is suitable for the analysis of conceptualisations of other diseases.

The article consists of three parts: a presentation of the analytic framework, a presentation of the AD conceptualisations of NIA-AA and the IWG, and an analysis of these conceptualisations through the framework.

## The analytic framework: nine structuring questions

The analytic framework consists of nine questions that enable and help structure the analysis of the “conceptual world” (Holstein 2000, p. 158) of new conceptualisations of diseases such as the IWG’s and the NIA-AA’s conceptualisations of AD. The questions focus on links and decouplings of the concepts of screening, diagnosis, pathology, disease, symptoms, and illness; their meanings and effects, and the conditions of emergence of these links/decouplings (Table 1). The analytic framework is also designed to help explore how specific decouplings put some “options into focus while obscuring others” (ibid.).

**Table 1:** The analytic framework: nine structuring questions

1. Is diagnosis decoupled from disease?
2. Is pathology decoupled from disease?
3. How does the perceived relationships (the links and decouplings) between diagnosis, pathology, and disease, when held together, affect the conceptualisation of disease, and what can be learnt from comparisons with older biological-physiological or normative conception of disease in this regard?
4. Is disease decoupled from symptoms?
5. What role is given to objective and/or subjective symptoms; and what role, if any, is given to first-person experiences, i.e. to illness, in the conceptualisation of disease?
6. What is the relationship between testing, screening and diagnosis; if a certain testing, under a specific disease conceptualisation, constitutes screening, what does that help achieve?
7. How can the above decouplings or linkages help enact some “spaces of possibilities” more than others, and what are the implications of this?
8. What do the above decouplings/linkages help achieve: for the individual, for society, for our understanding of human life, for clinics?
9. Under what conditions do the decouplings/linkages emerge: what makes them possible?

The first question addresses the decoupling or link between the concepts of diagnosis and disease. On the one hand, everyday language sometimes link “diagnosis” and “disease” closely together, as when people state that they have been diagnosed with Alzheimer’s disease. That which is diagnosed, in such a formulation, is a disease. On the other hand, the term diagnosis stems from the Greek *diagignoskein*, meaning “to discern” or “to know thoroughly” (Hofmann 2019, p. 1812). If diagnosis is understood to be discerning or learning that results in thorough knowledge, then that which is diagnosed need not be a (manifest) disease. This is a first possible decoupling: a decoupling of *diagnosis* from *disease*. Such a decoupling makes it possible to talk about the diagnosis of risk factors or indicators, as noted (and problematized) by Hofmann (2019). This first decoupling can be combined with a second decoupling, which the second question focuses on: the decoupling of *pathology* and *disease.* A consequence of this second decoupling is that a person who has been identified as having a certain pathology is not necessarily also identified as having a disease. Whether this is the case will depend on how pathology and disease are defined and related to (linked or decoupled from) each other.

Please note that a central analytic idea, upon which this framework is based, is this: by asking about and analysing linkages and decouplings between diagnosis and disease, and disease and pathology, it is possible to clarify how the concepts of diagnosis, pathology and disease are understood from within a certain conception of AD or another disease. This also means that the framework does not start by defining how the concept of disease is and should be understood but rather examines how, for example, the concept of disease is used and what it is taken to refer to *from within a certain conceptual world*.

Further, the third question asks how the perceived relationship between diagnosis, pathology, and disease, when held together, affect or help shape the conceptualisation of disease, and what can be learnt from comparisons with biological-physiological or normative conception of disease in this regard. This question is based on the idea that the decouplings and linkages, the relation or lack of relation between the concepts of diagnosis, pathology, and disease within a certain conceptualisation of AD or another disease does affect how disease is understood—and that applying this third question to a certain conceptualisation of AD or another disease helps clarify that conceptualisation.

The fourth and fifth questions address the conceptual triad of disease, symptoms, and illness. The fourth question asks whether *disease* is decoupled from *symptoms*. Not all conceptualisations of disease require symptoms to be manifest, and manifest symptoms can be perceived and experienced by the patients (often labelled as subjective symptoms) as well as observed by others, often measured with medical technology (i.e. symptoms from what is sometimes labelled a third person perspective). The fifth question explicitly asks about the role given to subjective symptoms and/or symptoms from a third-person perspective and the role, if any, that is given to illness in the conceptualisation of disease. The term “illness” is commonly used to refer to an experiential dimension from a first-person subjective perspective: it refers, then, to felt pain and suffering, felt bodily and/or mental alienation, or experienced incapacitation (see, for example, Carel 2008), and the choice to link or decouple disease from illness will affect the role that can be given to first-person experiences of illness in the diagnosis of the disease.

The questions in the framework enable the analysis of how conceptualisations of disease are parts of tangled conceptual webs of interrelated terms. The first five questions focus on diagnosis, pathology, disease, symptom, and illness because these are central to conceptualisations of disease, such as those suggested by the NIA-AA and the IWG, as the latter part of this article will show. However, other concepts could also have been included such as that of sickness. Central to sickness discussions are discussions of entitlements to treatment, economic support or exemption from work. However, while the understanding of sickness can be crucial to analyses of social welfare systems, and while not being deemed sick and hence not entitled to economic support can negatively affect one’s experience of being ill, I see the concept of sickness as less central to the conceptualisation of disease in the NIA-AA and the IWG.^2^ For this and for space reasons, I have not included a question about the relation between disease, illness, and sickness—though this is an important topic.^3^

The sixth question addresses the relation between testing, screening and diagnosis, and asks “if a certain testing, under a specific disease conceptualisation, constitutes screening, what does that help achieve?” While it could be held that whether a certain testing constitutes screening or not is or should be irrelevant for the discussion of conceptualisations of disease, this sixth question is based on the idea that particular conceptualisations of disease can open up for early testing also in forms that qualify as screening, and that whether they do so partly depends on decouplings and linkages discussed within the framework. In this way, also question six contributes to the clarification and analysis of conceptualisations of disease.

Medical screening is the term commonly used to describe a medical assessment that is offered by healthcare professionals to people who do not seek healthcare on the basis of symptoms. The UK National Screening Committee describes screening as a “public health service in which members of a defined population, who do not necessarily perceive they are at risk of, or are already affected by, a disease or its complications, are asked a question or offered a test to identify those individuals who are more likely to be helped than harmed by further tests or treatment to reduce the risk of disease or its complications” (UK NSC 2017). Further, opportunistic screening typically refers to screening of people for a certain disease, when they visit health-care for other reasons than those for which they are screened.

Finally, the analytic framework is designed to enable analysis of meanings and effects of specific conceptualisations of diseases, and conditions of emergence. For this reason, after having spelt out distinct decouplings and links as those above, three more questions are asked, which analytically cut across and build on the decouplings and linkages identified when answering the previous questions. Question seven asks how the decouplings or linkages help enact some “spaces of possibilities” more than others, and what the implications are, if this is the case. Question eight continues along related lines, but broadens the scope of the question, asking what the implications of the previous decouplings or linkages are and what these decouplings or linkages help achieve for the individual, for society, for our understanding of human life, and for clinics. Both these questions require reflection about what a set of decouplings and linkages makes possible, together. The ninth question asks a contextualising question, about the contextual factors that together can help make possible the types of decouplings, linkages, and conceptualisations that are suggested. The last three questions are important for the discussion of the “making up” of new people and related phenomena (Hacking 1985).

## Emerging conceptualisations of AD

An ambiguous relationship between clinical symptoms and neuropathology has characterised conceptualisations of AD during the twentieth century, and societal, scientific and technological changes have contributed to changes in conceptualisations (see, for example, Boenink 2016; Lock 2013; Ballenger 2006; Holstein 2000; Fox 1989, 2000). The dual focus on clinical symptoms and neuropathology was present already in Alois Alzheimer’s 1907 description a middle-aged woman who had symptoms of what was understood as presenile dementia, and in whom a *post mortem* biopsy revealed not only the plaques characteristic of presenile dementia but also “strange and noteworthy” neurofibrillary tangles (Alzheimer 1907, p. 147. My translation). A few years later Emil Kraepelin coined the term “Alzheimer’s disease” to describe a distinct form of middle-age-onset dementia. The patient’s age was used to differentiate between AD and what was called “senile dementia”, where the latter was perceived as (more or less) part of natural human aging (see, for example, Holstein 2000).

Diagnostic practices gradually changed from the 1970s onwards, when research showed that many older people had similar neuropathological tangles in their brains as persons diagnosed with AD (see, for example, Katzman 1976). This observation, in combination with sociocultural changes in the perception of cognitive decline that did not accept such decline as an inevitable part of aging (Holstein 2000; Ballenger 2006), led to the distinction between senile dementia (as part of old age) and AD (as a middle-age phenomenon) (Fox 2000) being abandoned. New terminology was introduced such as “Dementia of the Alzheimer Type” as a disease that was distinct from “normal’ aging” even though age was its most important risk factor (Holstein 2000, p. 158; Fox 1989). Removing the age criterion for a diagnosis of AD led to a rise in the number of people diagnosed with the condition (Ballenger 2006), at a time where sociopolitical movements brought together the US National Institute of Neurological and Communicative Disorders and Stroke (NINCDS) and the Alzheimer’s Disease and Related Disorders Association (ADRDA) in efforts to gain increased funding of biomedical research and the establishment of AD policies on the federal level (Fox 2000). The combined body, NINCDS-ADRDA, gradually managed to create a social movement around AD—and successfully argued that resources should be devoted to AD biomedical research (Fox 1989, 2000).

AD, the NINCDS-ADRDA stated, is a “brain disorder characterized by a progressive dementia that occurs in middle or late life” (McKhann et al. 1984, p. 939). While the NINCDS-ADRDA remained agnostic as to aetiology (McKhann et al. 2011; Frisoni et al. 2011), it outlined clinical criteria for the diagnosis of “possible AD” and “probable AD”, and stated that the diagnosis of “definite AD” was to be made if the clinical criteria for probable AD were present *together with* “histopathological evidence” from autopsy or, more rarely, biopsy (McKhann et al. 1984, p. 940). In this way, definite AD was still to be identified by clinical symptoms during life and by pathological findings in an autopsy or biopsy.

The recommendations of the NINCDS-ADRDA can be read partly as a contrast to and partly as a precursor to those of the NIA-AA and the IWG. The NINCDS-ADRDA called for more research into neurotransmitters and biomarkers, and the results of such biomedical research are the starting point for the work of the NIA-AA and IWG. Biomedical research has sought to characterise and identify the biological basis of the pathophysiological process of AD and has, more recently, focused on how the peptide Aβ and the microtubule-associated protein tau form AD-characteristic senile plaques and neurofibrillary tangles and contribute to neuronal loss and neural degeneration (see, for example, Kametani and Hasegawa 2018). Further, biomarker-based diagnosis of AD, brain imaging of amyloid plaques, and measures of the degree of tau and Aβ deposition in the cerebrospinal fluid (CSF) have been proposed as possible methods to increase the reliability of AD diagnosis.

However, the relation between AD biomarker-based diagnosis and a later manifest AD is debated. On the one hand, a consensus is growing that AD biomarkers and neurodegeneration do indicate AD-related changes in the brain, and that such changes can be detected several years before the manifestation of clinical symptoms (Karlawish et al. 2017). On the other hand, pathological brain changes and clinical symptoms do not always align with each other, which among others the NIA-AA acknowledges (see Sperling et al. 2011; Jack et al. 2018). Studies have shown that 10–30% of individuals who are clinically diagnosed with AD dementia have CSF AD biomarkers within the normal interval and no AD-related neuropathological changes at autopsy (see Jack et al. 2018; Nelson et al. 2011). Autopsy studies have also shown that only 30–40% of individuals who have cognitive impairment have AD-related neuropathological brain changes and “abnormal” amyloid biomarkers (Jack et al. 2018, p. 538; McKhann et al. 1984), and Jack et al. suggest that “cognitive symptoms are not an ideal way to define AD” (2018, p. 538). Biomarker-based AD diagnosis, they hold, is preferable. Taking this one step further, it has been suggested that screening for AD biomarkers in patients with “cognitive problems” in blood—which is easier to obtain than CSF—can be used in primary care (Blennow and Zetterberg 2018, p. 643).

The frameworks suggested by the IWG (Dubois et al. 2007, 2010, 2014, 2016) and the NIA-AA (Jack et al. 2011, 2018; Sperling et al. 2011; McKhann et al. 2011) give a central place to the use of biomarkers in the early diagnosis of AD, typically with the emphasis that such biomarker testing, so far, should be done for research purposes. The NIA-AA has also recently suggested that a strict demarcation be drawn between clinical symptoms and neuropathological changes in the definition of AD (Jack et al. 2018). The IWG, in contrast, generally maintains a dual clinical-biological focus in the diagnosis of AD (Dubois et al. 2010, 2014). Each group has refined and modified its conceptualisations and criteria over time.

### The framework of the IWG

The IWG has argued for a research framework of AD that “covers its full spectrum” (Dubois et al. 2010, p. 1118).^4^ Three aspects of the IWG discussions are noteworthy. First, the IWG differentiates between the “underlying pathology of the disease at the genetic, molecular, or cellular level”, which is termed “Alzheimer’s pathology” and may or may not be combined with clinical symptoms, *and* AD (Dubois et al. 2010, p. 1121; Table 1). The disease of AD is understood to be a “clinicobiological entity” (Dubois et al. 2014, p. 1118), and the clinical-biological is mirrored in the diagnostic procedure: both clinical *and *in vivo biological manifestations of the disease (such as biomarkers) must be assessed for a diagnosis of AD to be reached (Dubois et al. 2010, p. 1119; Dubois et al. 2014, p. 622). The clinical-biological foci also recur (in Dubois 2018: 1064), when it is stated that the diagnosis of AD requires “the presence of an appropriate clinical AD phenotype (typical or atypical) and a pathophysiological biomarker consistent with the presence of AD pathology”. (Even though, Dubois and collegues also write, in 2016, that “we may consider that AD exists and can be recognized before the onset of cognitive symptoms when there is little doubt about progression to clinical disease over a short period”, see Dubois et al. 2016, p. 298).

Second, the IWG outlines a set of stages of AD. “Two phases of the disease” “in the continuum” of AD are differentiated: a preclinical stage and a clinical stage (Dubois et al. 2016, p. 295; Table 1). The term “preclinical stage” (or “stages”) of AD, the IWG clarifies, should be used when at least one biomarker of Alzheimer’s pathology has been detected, *and* when the person being diagnosed has no clinical symptoms (Dubois et al. 2016, Table 1). Preclinical AD is to be identified based on the presence of one (at least) biomarker of Alzheimer’s pathology, and requires “the absence of clinical signs and symptoms of AD” (Dubois et al. 2016, p. 295).^5^ For a diagnosis of “clinical AD”, in contrast, both clinical symptoms and biomarkers for AD must be present. Clinical, symptomatic AD has also been further differentiated into two stages: “predementia AD” (with episodic memory impairment that does not yet affect daily living) and “AD dementia”. This makes it possible to argue, as the IWG did in 2014, that clinical AD can be diagnosed independently of AD dementia, as long as some clinical symptoms that are specific for the AD episodic memory profile are present, and biomarkers for AD have been detected (Dubois et al. 2014, p. 614).

Third, the IWG suggests that the diagnosis of AD be “uncoupled from a particular threshold of severity,” and that there is “no longer a need to anchor the diagnosis of AD to a dementia syndrome” (Dubois et al. 2010, p. 1120). This is a move away from equating AD with AD dementia. Clinical symptoms remain central to the diagnosis of AD, but it is sufficient that they are present: there is no threshold level of severity. For the IWG, this change “has the major advantage that there is no longer a reason to wait until patients have developed full-blown dementia or to exclude from diagnosis and treatment a large number of patients who lack functional disability yet express the disease” (Dubois et al. 2010, p. 1120).^6^

### The framework of the NIA-AA

The NIA-AA takes another conceptual approach. On the one hand, the NIA-AA shares the view that AD should be understood as a continuum along a “spectrum” (Sperling et al. 2011, p. 282, Jack et al. 2018, p. 537). In its recommendations from 2011, the NIA-AA also differentiates between stages: preclinical/presymptomatic AD (Sperling et al. 2011), mild cognitive impairment of AD (Albert et al. 2011), and AD dementia (McKhann et al. 2011; Jack et al. 2011, 2018). In more recent recommendations, the NIA-AA emphasizes that AD is a “continuous process” (Jack et al. 2018, p. 547). On the other hand, the NIA-AA recommendations have been described as based on a “dissociation” between neuropathological changes and clinical symptoms (Sperling et al. 2011, p. 283), and as adhering to a “biologically based definition of AD” (Jack et al. 2018, p. 536). The 2018 NIA-AA framework states explicitly that AD is a “biological construct” that refers to an “an aggregate of neuropathological changes” that can be detected solely by the detection of biomarkers (Jack et al. 2018, pp. 539, 538).

Further, in its 2018 recommendations, the NIA-AA distinguishes between a “disease” and a “syndrome”, and maintains that “dementia is not ‘a disease’ but a syndrome composed of signs and symptoms that can be caused by multiple diseases, one of which is AD” (Jack et al. 2018, p. 538). AD is understood as referring to specific neuropathological changes that are defined “in vivo by biomarkers and by post-mortem examinations, not by clinical symptoms” (Jack et al. 2018, p. 538), and clinical symptoms are downplayed in the diagnosis of AD. Cognitive impairment is understood “as a symptom/sign of the disease,” while AD is defined biologically (Jack et al. 2018, p. 555). The 2018 NIA-AA recommendations rightly note that this implies “a profound shift in thinking”, and a step away from its own earlier discussions (as in Jack et al. 2011), where “biomarkers were used to support a diagnosis of AD in symptomatic individuals, but the definition of AD was not divorced from the clinical symptoms (with the exception of the 2011 NIA-AA recommendations on preclinical AD)” (Jack et al. 2018, p. 538).

The 2018 recommendations advocate a “divorce” in which the conceptualisation of AD should be set apart from clinical symptoms and clinical criteria (Jack et al. 2018). The term AD is to be “reserved” for persons in whom AD neuropathological changes and AD biomarkers are present (Jack et al. 2018, p. 541). Further terms are introduced: “Alzheimer’s clinical syndrome” is to be used for the clinical syndrome (Tables 2, 3).

**Table 2:** Simplified representation of the terminology suggested by the IWG (based on Dubois et al. 2014, 2016)

*Alzheimer’s pathology* A pathology identified based on the presence of AD biomarkers or other pathophysiological markers	*Alzheimer’s disease* A clinical-biological entity
*Preclinical AD* A stage of AD in which no clinical symptoms/clinical phenotype are present. Preclinical AD is identified on the basis of biomarkers of AD pathology only	*Clinical AD* Stages of AD where clinical symptoms/clinical phenotype and AD biomarkers are present

**Table 3:** Simplified representation of the terminology suggested by the NIA-AA (based on Jack et al. 2018, pp. 541, 547, 549)

*Alzheimer’s disease* A biological construct. AD “refers to Aβ plaques and pathological tau deposits, defined in vivo by abnormal biomarkers of Aβ and pathological tau” (Jack et al. 2018, p. 541).	*Alzheimer’s continuum* Involves a set of stages, from preclinical AD to AD dementia. Individuals at these stages have differing levels of AD biomarkers, and can vary from being “cognitively unimpaired” to having severe dementia symptoms	*Alzheimer’s clinical syndrome* The clinical syndrome

Even though they suggested a divorce between neuropathological changes and the clinical syndrome, the 2018 NIA-AA recommendations offer a system of severity stages based on biomarkers *and* the severity of cognitive impairment (Jack et al. 2018, p. 540). Thus, symptoms and their severity become relevant in the 2018 NIA-AA framework, to be used in a staging scheme from “cognitively unimpaired” to dementia (Jack et al. 2018, p. 549).

The 2018 NIA-AA framework contains several such staging schemes, and further details of the framework are not discussed here. However, the NIA-AA 2018 recommendations contain tables that bring together biomarker profiles and syndromal stages to enable the assessment of “risks of short-term cognitive decline” (Jack et al. 2018, p. 549). The recommendations also state that these stages do not describe an aetiology of AD, but “only severity of cognitive impairment” (Jack et al. 2018, p. 546). At stake, here, is a dual movement. On the one hand, the NIA-AA suggests that the neuropathological changes of AD (with biomarkers being understood as proxies for these changes) be split from clinical symptoms. This split is motivated also in earlier NIA-AA statements, when the view that there need not be a “close correspondence between clinical symptoms and the underlying pathology” is promoted (Jack et al. 2011, p. 258). On the other hand, efforts are made to offer routes—schemes—that bring together biomarker analysis and the severity of cognitive impairment for certain kinds of assessments of risks. These schemes have room for symptoms to be considered.

## Analysing the NIA-AA’s and the IWG’s AD conceptualisations

### Diagnosis, pathology, disease, and symptoms

Is diagnosis decoupled from disease? This is the first question in the analytic framework, and it centres on the possible decoupling of *diagnosis* from *disease*. This decoupling can take place if diagnosis is understood to be discerning or learning that results in thorough knowledge. If this is the case, then that which is diagnosed need not be a disease. It can also be the “diagnosis of risk factors” and the “diagnosis of indicators” for a disease. As seen, this first decoupling can also be combined with a decoupling of *pathology* and *disease*, and the second question in the framework asks about this second decoupling. If pathology is decoupled from disease, it would mean that a person who has been identified as having a pathology does not necessarily also have a disease. Whether this is the case will depend on, among other things, how the notion of disease is understood.

If the decouplings of diagnosis from disease and pathology from disease are combined and applied to AD discussions, this opens up for a reasoning in which that which is diagnosed can *both* be precursors for AD (based on an identified pathology) *and* AD. This expanded use of the term “diagnosis” can be contrasted, as Hofmann (2019, p. 1814) notes, with a narrower use in which the term is used solely for the diagnosis of “manifest disease and suffering”.

In the description of the analytic framework, I stated that the position one takes regarding decouplings between pathology and disease can depend partly on whether one adheres to biological-physiological conceptions or normative conceptions of disease. This can now be explained in some more detail, and applied to the discussion of the NIA-AA’s and the IWG’s conceptualisations of AD.

Biological-physiological conceptions of disease identify a state, ratio or level of an entity or biological/physiological function as statistically normal, and identify deviations from this normalcy as subnormal or abnormal functioning. The notion of disease, in such conceptions, refers to or is identical to the subnormal or abnormal functioning. Christopher Boorse’s oft-quoted conception of disease can be used as an example here (1977, 2014). For Boorse, diseases are “internal states that interfere with functions in the species design” (1977, p. 558). Disease is a matter of the subnormal functioning of an organ or some other part of the body, where the term “subnormal functioning” implies that the organ or part of the body functions at a level below that which is statistically normal for the species, in the specific reference group or class (specific to, for example, age or sex). Subnormal functioning is a functioning that is “below the mean of function” in “a typical environment” (Boorse 2014, p. 684).

The conceptualisation of AD as a physio-pathological entity put forward by the NIA-AA squares well with a biological-physiological conception such as that of Boorse, given its focus on abnormal functioning on the molecular level. AD, according to the NIA-AA conceptualisation, refers to specific neuropathological changes, and is “defined” by abnormal levels of the biomarkers pathological tau and Aβ (Jack et al. 2018, pp. 539, 541). The tau biomarker indicates a subnormal functioning of the tau protein.

From within the NIA-AA conceptualisation, that which is diagnosed is AD as the biological construct that refers to neuropathological changes. This construct refers to a certain pathology. This is to couple *diagnosis* with *disease*, *disease* with *pathology*, and to understand *disease along the lines of biological-physiological conceptions of disease*. That which is diagnosed in this reasoning is a disease—AD—where AD refers to neuropathological changes. According to the conceptualisation put forward by the NIA-AA in 2018, one cannot have AD without Alzheimer’s pathology, nor Alzheimer’s pathology without AD. However, another decoupling seems to take place: a decoupling between *disease* and *symptoms*. Symptoms become an optional extra that one may or may not have when one has been diagnosed with AD.

One more aspect of the biological-physiological conception of disease proposed by Boorse is relevant to my reasoning. Boorse considers diseases to be value-free on a basic level, and states that his conception requires “no value judgement about what forms of human life are admirable or desirable” (1977, p. 571). He goes on to qualify this statement. While arguing that no value judgement is needed for the diagnosis of disease where the “abnormal”, in the sense of the pathological, has been identified, he acknowledges that evaluative dimensions are present in clinical practice (“Should this disease be treated or not?”). Such evaluation is also manifest when decisions are made about “*therapeutic normality”,* as the “absence of a diagnostic abnormality worthy of treatment” (Boorse 2014, p. 685, italics in the original). For Boorse, the evaluation is part of what he terms “disease-plus” conceptions (2011, p. 28). The evaluative part arises as an addition, typically at the clinic, in order to decide what to do, whether—and if so how—to treat the patient.^7^

The NIA-AA 2018 suggestion is both similar and different in this regard. On the one hand, according to the terminology used, AD as a disease is not dependent on symptoms that makes it unwanted or undesired. The NIA-AA 2011 suggestion, as aptly noted by Schermer and Richard, “reflects the pathologist’s and [biomedical] researcher’s perspective”, as does Boorse’s theory (Schermer and Richard 2019, p. 141). On the other hand, however, the NIA-AA 2018 framework includes a severity stage-risk table that (while perhaps intended to be descriptive) implies some risk assessment, and risks, after all, are termed “risks” because the eventuality is understood to be negative.

The 2018 NIA-AA framework, in its systems for “staging severity,” includes grades of severity that are based on biomarkers and grades of severity of cognitive impairment (Jack et al. 2018, p. 540). The framework argues for a biological-pathological understanding of AD, but leaves room for the assessment of risks, and risks has connotations of something negative. The evaluative language is also present when the NIA-AA states, for example, that “the risk of short-term cognitive decline increases continuously with worsening (N) biomarkers” (where N stands for neurodegeneration/neuronal injury) (Dubois 2018, p. 542). Risk assessment, arguably, depends on values—that which is assessed is probability of some negative effects or future negative scenario. It can be discussed whether such risk assessments are examples of something akin to Boorse’s “disease-plus” idea. However, something partly different seems to be at stake in the NIA-AA 2018: the severity stages may not be defined in order to assess how to treat the patient, but to provide an independent assessment that helps to specify the severity of someone’s AD for classification purposes.

Further, the NIA-AA recommendations do not give room to a discussion of the role of experiences of possible *symptoms*—which I will come back to. The discussion omits also the question of whether receiving a diagnosis of AD, as specified by the NIA-AA recommendations, can result in felt harm or suffering. This may occur, for example, if the diagnosis results in severe and persistent worry about AD.

The IWG conception of AD, in contrast, has an evaluative and normative dimension, as have normative conceptions of disease. A normative conception understands disease in relation to human values, needs, and norms, and acknowledges that value judgements are made in the very conceptualisation of disease.^8^ Disease, in this understanding, describes a state of affairs that is undesirable, unpleasant, or unwanted—and that calls for action. Various such conceptions exist, such as the conception offered by Lennart Nordenfelt (1987, 2014). For Nordenfelt, disease is “defined as a state or process that tends to negatively affect its bearer’s health” (2014, p. 25). Health is understood in holistic terms, as a function of a person’s abilities to achieve her or his vital goals and act intentionally (Nordenfelt 1987, 2014). Health is compatible with the presence of disease, in such a case there arises “the probability” of limitations to one’s agency or negative sensations. If one has a disease, according to Nordenfelt’s conceptualisation, “it is plausible to say that there is a high risk” of having some negative sensations or other negative consequences (Nordenfelt 2014, p. 27).

Further, in a normative conception of disease, symptoms must have negative or probable negative implications for what one can do, or they must be experienced as negative. “Negative” in this context implies, for example, (at least a basic level of) harm, suffering or limited capacities. As Nordenfelt stated: if “there is no risk of suffering or disability at all, it is legitimate to ask what reason there would be for calling the state a disease in the first place” (2014, p. 27). A normative conception of disease requires at least a probability that symptoms negatively affect one’s abilities to achieve vital goals, and/or that such symptoms are experienced as negative. Symptoms without negative sensations or implications are not sufficient.

The IWG 2010 and 2014 approach, which requires symptoms to be present for a diagnosis of AD to be given, is compatible with this understanding, as also discussed by Schermer and Richard (2019). Yet, however, it can be questioned whether it is a clear-cut example of a normative conception of disease. As will be unpacked below, the IWG’s way of talking about the diagnosis of preclinical AD opens up for discussions of the meaning of “probable” negative implications, of the threshold of when possible negative implications get to qualify as probable, and whether—if negative implications of preclinical AD cannot justifiably be assessed as probable—the IWG’s conceptualisation of preclinical AD exemplifies normative conceptions of disease. I will take this reasoning in some steps:

The IWG 2010 and 2014 conceptualisation requires that a patient has biomarkers for AD and symptoms, in order for a diagnosis of AD to be reached. Biomarkers alone can show whether someone has Alzheimer’s pathology or not, but this is not sufficient for a diagnosis of AD. As seen, under this IWG conceptualisation, Alzheimer’s pathology refers to biological, pathological changes that “can exist without the concomitant clinical manifestations of AD”, while a diagnosis of AD requires that someone also has clinical symptoms (Dubois et al. 2010, p. 1118). For the IWG, someone can have Alzheimer’s pathology and be at risk for AD, without receiving a diagnosis of AD. Instead, such a person would be diagnosed with preclinical states of AD (see Dubois et al. 2014, p. 620). In this way, “diagnosis” under the IWG conceptualisation may be used when that which is diagnosed is not AD (since AD is repeatedly described as a clinical-biological entity) but preclinical AD, based on Alzheimer’s pathology.

The IWG seems, then, to decouple *diagnosis* from *disease* and to differentiate between *pathology* and *disease*. *If* symptoms are present, a diagnosis of AD may be reached, in the IWG terminology, but the term “diagnosis” is used not solely to denote the diagnosis of a disease (i.e. one with symptoms). Further, everyone diagnosed with AD has Alzheimer’s pathology, according to the IWG, but not everyone with Alzheimer’s pathology has AD, since (or as long as) AD is considered to be a clinical-biological entity, in contrast to preclinical AD.^9^ While the last point is somewhat paradoxical (to be discussed below), the approach taken by the IWG to AD does mean that *symptoms* and *disease* are kept together. This makes sense, given a normative conception of disease. In such a conceptualisation, a disease is only present if negative effects are experienced or probable.

The IWG emphasises that symptoms do not need to reach a *specific severity* for a diagnosis of AD to be made, only that symptoms need to be present. As formulated by the IWG: the diagnosis of AD is “uncoupled from a particular threshold of severity,” and there is “no longer a need to anchor the diagnosis of AD to a dementia syndrome” (Dubois et al. 2010, p. 1120).^10^ This opens for different interpretations in relation to normative conceptions of disease.

If it is enough for normative conceptions of disease that the disease probably limits one’s agency or probably brings negative sensations, then a diagnosis of preclinical AD can be understood as exemplifying such a disease conception. However, the inclusion of “probability” (as in “probable negative effects”) brings new difficulties: what percentage of risk is needed to state that preclinical AD will *probably* result in negative implications in terms of harm, suffering or limited capacities? This makes the terms “probable” or “risk” central, as can also be seen in Dubois et al.’s discussion (2016) of AD, when they discuss an “arbitrary dichotomy” between “an already developed AD pathology” and “a situation at risk of AD mainly in asymptomatic individuals” (who exhibit “an isolated brain amyloidopathy […] or tauopathy” (Ibid.).

Here, I contend, it needs to be asked whether preclinical AD can and should be understood as complying with a normative conception of disease if it: (i) is not perceived as a disease (because of the lack of clinical symptoms), and (ii) if “probable negative effects” (in the sense of developing symptoms) may be long delayed or, indeed, never arise because this person dies, for other reasons, before this happens? Further, if one follows the IWG 2010 and 2014 understanding of AD as a clinical-biological entity, then “preclinical AD” is an oxymoron: “preclinical Alzheimer’s disease”, to spell out the abbreviation, does not qualify as a disease. A disease, as defined by the IWG, requires clinical symptoms. A possible way to understand this conundrum is this: if one adheres to a normative disease conception and at the same time wants to acknowledge a preclinical stage of AD, identified on the basis of AD biomarkers only, established terms such as “disease” become slippery. This slipperiness has found its way into the IWG formulations.

Further, given the IWG’s terminology, we can ask about the consequences of being diagnosed with preclinical AD. Does this diagnosis result in patients being more self-aware? Will they interpret memory lapses as subjective symptoms? Research on the lived experience of being at risk for Huntington’s disease (while not knowing whether one has inherited the gene for this disease) has shown that an awareness of the risk can “take over and transform” everyday life. Everyday events, such as dropping a mug or stumbling, are interpreted as symptoms, by some persons (Hagen 2018, p. 78). In the context of the IWG conceptualisation of AD, how would memory lapses be perceived? If they are carefully noted due to the preclinical AD diagnosis and interpreted as very early symptoms of AD by the patient and the doctor, could the very process of diagnosing someone with preclinical AD contribute to this person qualifying for the diagnosis of AD? Whether this occurs may depend on how symptoms are understood and evaluated when determining a diagnosis of AD. (Do they need to be of a certain severity and frequency to qualify as symptoms that are deemed relevant for the diagnosis?). However, even if subjectively experienced memory lapses are not symptoms in the sense required for the diagnosis of AD, the existential dimensions of being diagnosed with preclinical AD must be taken seriously.

The links and decouplings that one makes can be informed by one’s stand in the debate between biological-physiological conceptions and normative conceptions of disease: the links and decouplings may make more or less sense, depending on this stand. However, the links and decoulings can also make more or less sense depending on how one perceives the purpose of a certain conception of disease. For example, if the purpose is to use the conception as a basis in a clinical setting, when diagnosing patients with manifest AD, it can make less sense to decouple symptoms from disease than if the purpose is to use the conception in a research project that seeks to identify AD biomarkers and cluster research participants into different groups based on the presence of different AD biomarkers.

Further, certain decouplings open or close discursive spaces. If it is considered that that which is diagnosed should always be a disease, and if diseases are understood along the lines of the normative conceptions of disease, then it can make more sense to talk about the identification of biomarkers that are characteristic for AD than about the diagnosis of a preclinical disease. If diseases are understood along the lines of biological-physiological conceptions, then it can indeed make sense to diagnose someone with AD based on the identification of biomarkers characteristic for AD. In any case, the choice of decouplings and links between diagnosis and disease, and disease and pathology are not neutral in the light of the way in which the formulation “I have been diagnosed with…” can carry connotations of stability and severity, and function as a powerful social and medical tool (cf. Jutel 2009).

Further, how one understands diagnosis and whether diagnosis is decoupled from disease are also relevant in discussions of overdiagnosis. If overdiagnosis is understood to be “unwarrantedly giving a person the label of disease”, which occurs when “we connect information about an identified entity to disease without knowing whether it will result in manifest disease and suffering” (Hofmann 2019, pp. 1812, 1813), then the analysis also plays out differently in relation to the recommendations of the IWG and the NIA-AA. The risk for overdiagnosis may seem to be high when the NIA-AA conceptualisation is applied. Overdiagnosis typically means that individuals who would otherwise *not* have been given a diagnosis of a particular disease because they would never experience any symptoms of the disease are, nevertheless, given this diagnosis. It is difficult to see how this can be avoided with the NIA-AA conceptualisation. On the basis of the IWG 2014’s conceptualisation, in contrast, the NIA-AA conceptualisation can be criticised precisely on the topic of overdiagnosis: from within this IWG conceptualisation, it may be argued, the NIA-AA identifies precursors of AD (where someone is “at risk” for AD), not AD. This means that overdiagnosis will occur in the cases in which Alzheimer’s disease (according to the NIA-AA’s use of the term) that has been detected from the presence of biomarkers does not result in clinical symptoms and manifest disease and suffering.

### Disease, symptoms, and illness

The fourth and fifth questions in the framework address the relation between disease and symptoms (is disease decoupled from symptoms?), the understanding of symptoms and the role, if any, given to illness in the conceptualisation of disease. This has already been briefly discussed above. As seen, the term illness, in this article, refers to an experiential dimension from a first-person perspective that can include felt symptoms of different kinds, felt bodily and/or mental alienation and experienced incapacitation.

Following the NIA-AA terminology, AD is diagnosed on the basis of neuropathology and the presence of biomarkers. A person can have AD without having any symptoms, and first-person experiences of suffering, alienation or inability are not required. In this sense, while the NIA-AA unites diagnosis, pathology and disease, these are *decoupled* from *illness*. Even so, felt suffering and felt incapacitation can become relevant, following the NIA-AA understanding, in relation to the stages of the severity of cognitive impairment. Whether this is the case is an open question, since it is not clear what role the NIA-AA gives to lived experiences.

The IWG, in contrast, makes an explicit acknowledgment of the role of clinical symptoms, and thus may seem better able to accommodate concerns with illness. This, however, is not necessarily the case. Again, if clinical symptoms are integrated into the diagnostic procedure, this need not imply that the patients’ own felt experiences are rendered central. Various tests are available to score the cognitive abilities of patients in ways that focus on what they can do (sometimes referred to as “objective measurements”, or measurements from a third-person perspective), and these need not include the experience of cognitive impairment from a first-person point of view. Following the IWG conceptualisation, someone can be diagnosed with AD only if she or he also has clinical symptoms. The role of the *felt* experience of these symptoms—which may be episodic and temporarily restricted—for the diagnosis seems to be more of an open question.

### Screening and diagnosis

What is the relationship between testing for AD biomarkers, screening and the diagnosis of AD under the NIA-AA and the IWG reasoning, and if a certain testing, under a specific disease conceptualisation, constitutes screening, what does that help achieve? This is the sixth question within the framework, applied to these conceptualisations of AD.

The term screening isn’t used in the NIA-AA recommendations, with the exception to Jack et al. (2018, p. 555), where AD biomarker tests are described as likely to have a “screening role” in the future (see next section). However, through its specific decouplings and linkages, the NIA-AA conceptualisation opens up for a diagnosis of AD to be made very early—years before any symptoms are present. Indeed, one may ask, could anything be earlier than the early testing of AD based on biomarkers and neuropathology, i.e. earlier than a test that some persons take at a stage when they have no symptoms and for this reason do not “necessarily perceive they are at risk” of AD (as in the UK screening formulation)? To say the least: such a testing is a very early testing, and the NIA-AA conceptualisation *opens up* for such very early testing. This leads to questions of the role of screening under the conceptualisation of the NIA-AA, as a practice that typically takes place early and targets groups within a population that have no symptoms. *If* this very early test is offered to everyone above a certain age, when they visit primary care for other reasons, it is a candidate for the label of opportunistic screening. Or, with another example, if AD biomarker testing is offered to people above a certain age interval who have had no symptoms of AD dementia and take part in research projects on AD biomarkers for other reasons, also this practice may qualify as a case of opportunistic screening (though limited to the participants in the research project). In these cases, and under the NIA-AA conception, a positive test result for AD biomarkers would be the basis for AD diagnosis, and it would not be based on symptoms but offered to people within a certain group who do not perceive themselves at risk, necessarily.

In this way, conceptualisations of AD can open up for early testing practices for AD that qualify as screening, and whether they open up for this partly hinges on decouplings and linkages discussed within the framework: through the decoulings and links that positions early testing of AD based on biomarkers and neuropathology as central to the diagnosis of AD. The development of fast and easy to use AD biomarker tests can help open up for opportunistic AD screening of asymptomatic persons—even if this is not the NIA-AA’s intention.

In the IWG case, Dubois et al. describe a conceptual shift that has led to a “disease model that begins with risk factor assessment (directed at the potential for primary prevention), advances to screening (for early detection and early intervention of disease—secondary prevention), and leads to treatments and monitoring of treatment effects” (Dubois et al. 2016, p. 294). This approach, they add, includes “screening with tests with high sensitivity, lower specificity, and low costs, to those with higher specificity and value with potential for longitudinal quantification” (ibid). The IWG does not discuss screening any further, and two interpretations are possible. Either screening is temporally inserted between the diagnosis of preclinical AD and the diagnosis of clinical AD, as a screening for early symptoms of clinical AD; or (arguably a more stretched interpretation) that AD biomarkers are screened for, which makes screening for preclinical AD (where screening could mean that a biomarker test is offered to a part of a population who do not perceive themselves as affected by AD) that which result in the diagnosis of preclinical AD (where this diagnosis is given based on the result of the same biomarker test).

Irrespective of when screening takes place under the IWG reasoning, the conceptualisations of AD, in the IWG and in the NIA-AA, have implications for how to understand the temporal relationship between screening, testing for AD biomarkers and the diagnosis of AD. Further, much screening ethics have examined population-based screening practices. National committees that assess such screening practices including ethical aspects of them are established in many countries, while ethical guidelines for opportunistic screening are much more rare. If some AD conceptualisations encourages practices of early testing for AD, which may then be performed in ways that qualify as opportunistic screening, then it is high time that ethical guidelines also for opportunistic screening practices are developed—akin to those applied to population-based screening practices.

### Spaces of possibilities and other implications

Question seven and eight require reflection about what the decouplings and linkages makes possible, together. They ask how the decouplings or linkages can help enact some “spaces of possibilities” (Hacking 1985, p. 165) more than others, and what the decouplings or linkages can help achieve, more broadly, for people as individuals, for society, for our understanding of human life, and for clinics.

What spaces of possibilities do the NIA-AA and the IWG make possible? As already discussed, the decouplings and linkages of the NIA-AA help to enact spaces of possibilities in which the detection of biomarkers can be given priority. This becomes particularly possible thanks to the specific linkage of pathology and disease and the decoupling of disease and symptoms in the NIA-AA’s recommendations. If the NIA-AA conceptualisation were to become *the* conceptualisation that is used in research and at clinics, and if clinics were to align their work with these recommended conceptualisations and categories of different stages of AD, this would help to give priority to biomarkers, and put preclinical AD into a category of its own. This is also a possible outcome if the IWG conceptualisation were to become *the* established conceptualisation, even though the IWG’s focus on a dual clinical-biological understanding of AD leads to a less intense focus on biomarkers. From within the IWG conceptualisation, the enacted spaces of possibilities include spaces in which symptoms are still regarded as central to the diagnosis of AD (though not to that of preclinical AD).

It is important to note, as shown by Boenink, that the NIA-AA’s guideline can function both as “gatekeepers and trailblazers” (2017, p. 224). The NIA-AA has been cautious towards the introduction of AD biomarkers tests into clinical practice, yet their guidelines “can create a world which is prepared to include certain technologies in the future, even while advising against introducing the same technologies into current clinical practice” (Boenink 2017, p. 225). The NIA-AA does discuss AD biomarkers as part of future practices, as when stating that “in the future, less-invasive/less-expensive blood-based biomarker tests along with genetics, clinical, and demographic information will likely play an important screening role in selecting individuals for more-expensive/more-invasive biomarker testing” (Jack et al. 2018, p. 555). In this way, and as put by Boenink, “these guidelines pave the way for a smooth introduction of biomarker tests later on. They act as trailblazers, even when they are also gatekeepers” (2017, p. 224).

If preclinical AD is made a category of its own, this have specific effects. For example, both the NIA-AA and the IWG use the term “preclinical AD” and suggest that preclinical AD can be diagnosed based on biomarkers only. Such a recommendation for a diagnostic procedure mobilise biomarkers as central in the discourse, and as offering conditions for the phenomenon that Hacking termed the “make up” of new people (Hacking 1985). New conceptualisations and categories, Hacking argued, could help to enact new “spaces of possibilities”, people can be placed into new categories, understand themselves in new ways through them, mobilise themselves into new social groups, and help to modify the initial categories (Hacking 1985, p. 165). In the present discussion, new categories of people, such as those with preclinical AD, come to exist, which can feed into the ways people make use of and understand AD conceptualizations. The new categorisation can affect behaviour, such as calls for AD biomarker testing and further follow-ups. At stake, to use Hacking’s formulation, are “looping effects”: new categories, new “modes of description come into being, new possibilities for action come into being in consequence” (1985, p. 166).

With the availability of categories of preclinical AD, more people can come to ask for tests for preclinical AD, use this category as part of their self-understanding, which can further promote calls for such testing—and promote a shift in the understanding of AD towards an understanding that sees preclinical AD as a disease for which treatment should be developed and given. Indeed, the availability of biomarker-based diagnosis for AD has already helped to create a new category of people: people with preclinical AD (see, for example, the Waters’ Biomedical Website which asks “Do you have Preclinical Alzheimer's Disease?”, followed by the statement: “47 Million of us [in the USA] do!” (Waters 2018).^11^

Conceptualisations of preclinical AD and the looping effects can also contribute to a *pathologising* and a *diseasisation* of human life. A pathologising of human life occurs if it comes to be perceived as normal that people are identified as having a pathology before they have any symptoms. A pathologising of human life can be understood as part of a larger tendency of biomedicalisation. As suggested by Clarke et al. (2003, p. 162), biomedicalisation refers to “the increasingly complex, multisited, multidirectional processes of medicalisation that today are being both extended and reconstituted through the emergence of social forms and practices of a highly and increasingly technoscientific biomedicine.” Clarke and colleagues suggest that, in contrast to medicalisation and its focus on “control over medical phenomena” that were previously not seen as medical, biomedicalisation practices “emphasize *transformations* of such medical phenomena and of bodies, largely through sooner-than-later technoscientific interventions” (Clarke et al. 2010, p. 2).

The IWG’s understanding of preclinical AD—through its decoupling of diagnosis from disease and differentiation between pathology and disease—may open up for such a pathologising, without this being the intention of the IWG. Under the IWG, it is possible to be identified as having a pathology before one has any symptoms. In this way, the new conceptualisations of AD underscore concerns over biomedicalisation. If the category of preclinical AD becomes established, parts of life that have not been perceived as falling within the domain of medicine can start to do so.

Further, however, if the NIA-AA conceptualisation and categories come to be used, it will not be necessary to manifest clinical symptoms of AD to be given a diagnosis of AD—and this can help transform people’s understanding of the role of symptoms for disease, though a technoscientific process of biomarker-based diagnosis. Symptoms, following the NIA-AA, are not needed for the diagnosis of AD. Indeed, because the NIA-AA holds together pathology and disease, more is at stake than a pathologisation in which having pathologies—at a genetic, molecular, or cellular level—may become normal. The NIA-AA conceptualisation, if accepted, may lead to what can be called a *diseasisation,* in which preclinical non-symptomatic conditions are understood to be diseases. Given the NIA-AA’s vocabulary, if I test myself for AD biomarkers, I will not only learn about underlying pathology that may or may not manifest in symptoms: I will learn that I have, already, the disease of AD.^12^ This, then, is different from the already common situation where someone takes pills in order to minimize the risk for a disease (as when someone takes a drug to decrease blood pressure, as a preventive measure, to lower the risk for a heart attack). Such a shift in conceptualisation, and the diseasisation, is of no small consequence: it can become a powerful tool for people diagnosed with preclinical AD to put pressure on different social and medical actors to prioritize the development of treatment and for proponents of technoscientific interventions of AD to put pressure on politicians to fund more research for the development of drugs or other interventions—arguing that they have now been diagnosed with this disease. Such development can of course be beneficial and central to future treatments of AD, and I am by no means arguing against the value of such treatment. However, the new conceptualisations can also contribute to a shift in shared understandings of what it means to have a disease, and to a diseasisation of human existence where to be diagnosed with diseases without having any symptoms comes to be perceived as a responsible way of acting, by patients and doctors. This can also help open up for screening practices: in such a future scenario, to screen for preclinical AD might be one route to a later diagnosis of preclinical AD. Indeed, a number of trials are already on-going with the aim of developing tests for AD biomarkers in blood samples, and such blood-based “screening biomarkers for AD can meet the scalability needs required for primary care settings and even for the broad population-based screening approach that may evolve with the advancing innovative precision medicine framework”, suggests Hampel et al. (2018, p. 641).

Possible implications of the category of preclinical AD, should it become established, has been discussed also elsewhere (cf. Boenink 2017). The analytic framework, however, helps clarify how implications such as that which I have labelled as diseasisation hang together with specific decouplings and linkages between—in this case—pathology and disease.

Further, the current situation in which there is no cure and only modest treatment for AD sharpens ethical concerns if or when preclinical AD were to become an established category. Such an establishment could help create an existentially-temporally condensed situation in which patients, when diagnosed with preclinical AD, are made to consider their possible future(s), should the preclinical AD develop into AD dementia—and make sense of the uncertainties involved (since not everyone with preclinical AD develops AD dementia). If the NIA-AA conceptualisation becomes the established one, we must ask how we are to live with the implications of diseasisation, should one’s biomarker test be positive. For healthcare professionals, the choice of whether to offer detection of AD biomarkers when no cure and little treatment for AD is available is intertwined with the question of what existential issues and decisions healthcare systems help to make central for patients and potential patients. The support available for those who test positive is also a consideration, though not a new challenge. What is partly new in dementia settings, however, and a question to address for healthcare professionals who offer biomarker testing, is whether their way of talking about, framing and explaining such testing contributes to diseasisation, and whether this should be seen as troubling or not—for patients, the healthcare system, and society.

### Conditions of emergence

The last question, question nine, asks under what conditions of the decouplings/linkages emerge, thereby acknowledging that decouplings and linkages do not take place in sociocultural vacuums. The description of past work on the establishment of the NINCDR-ARDRA and its recommendations (Fox 2000) makes it clear that AD conceptualisations emerge as possible, and become accepted, in specific times and places. These are times and places at which scientific and societal shifts have taken and are taking place. This is key also to understanding the NIA-AA and the IWG recommendations. They become possible and acceptable given specific biomedical developments and societal understandings of risk and aging combined with calls by specific stakeholders for a rethinking of past conceptualisations. In this regard, it is relevant that the NIA-AA invited a group of people to draw up the recommendations, and gave them this task. Further, the political situatedness of the NIA-AA means that its recommendations are not just any recommendations, and concerns that these recommendations have a “powerful impetus to the spread of biomarkers into diagnostic practice” (McCleary et al. 2019, p. 176) must be understood in this light.

To ask about conditions of emergence of conceptualisations of AD is to ask about how societies and scientific understandings co-emerge, and about possible alliances between stakeholders that enable shifts in conceptualisations of AD. This ninth question is needed in order to bring out the role and distribution of power when conceptualisations are developed and established, as a way to contextualize and make sense of shifts in conceptualisations. As question seven and eight, this question invites discussions about implications of the conceptualisations, this time in the form who wants them, and who stands possibly to gain and who to loose from the specific decouplings and linkages within them.

## Summary and concluding remarks

Past research has emphasised the need to discuss what AD conceptualisations can help to achieve, examined the goals of AD conceptualisations, and discussed the possible effects on individuals and societies (Schermer and Richards 2019; Boenink 2016; Karlawish 2011; Holstein 2000). This article adds to such reasoning through its attention to conceptual worlds, their meanings and implications, and it proposes a framework to use. It contributes tools for the analysis of how different conceptualisations help to direct the spotlight. Engaging with the questions in the framework also reveals the ways in which the conceptualisations of AD by the IWG and the NIA-AA are new, and the ways in which they are not. The analytic framework also targets what spaces of possibilities specific conceptualisations (and their decouplings and linkages) open to. When applied in particular to the NIA-AA’s conceptualisation, it shows how the category of preclinical AD can result in a new category of people, how people can understand themselves in new ways through this category, mobilise themselves into new social groups. Further, the NIA-AA’s conceptualisation can contribute to a diseasisation and a shift in shared understandings of what it means to have a disease. The article acknowledges that if AD is used to denote that which is not manifest disease but neuropathology, this is a radical shift in the understanding of this disease, with ramifications beyond the geriatric ward.

## Footnotes

1 The analytic framework is not designed to offer a normative assessment or justification of whether a certain conceptualisation should be deemed ethically unacceptable or not. However, such an assessment could be a second step, after the kind of analysis that the framework enables. This would require an analysis of its own, and will not be discussed in this article.

2 Further, in many welfare states, social support is given after someone has been given a diagnosis, and the diagnosis, rather than someone being deemed sick, serves as the basis for decisions regarding social support.

3 I could also have included the concept syndrome in the framework, but I have chosen not to do so. The concept of syndrome is often used to refer to collection of symptoms—a group of symptoms—associated with a specific disease, though this is not always the case. Within the analytic framework, I have chosen to ask about symptoms instead of syndromes, because the concept of symptoms and the distinction between subjective/objective symptoms allow me to unpack the role given to different kinds of symptoms in a way that the concept of syndrome is less apt to do.

4 The IWG does not use the formulation of “spectrum” to define “subthreshold cases” where symptoms are present but below established cut-off values. Instead, the concept of spectrum seems to be applied to the severity of symptoms and quantity of biomarkers along a temporal line: symptoms typically aggravate over time.

5 The FDA-NIH Biomarker Working Group understands biomarker as “a defined characteristic that is measured as an indicator of normal biological processes, pathogenic processes or responses to an exposure or intervention” (quoted in Cardiff 2018, p. 214). However, as shown by Cardiff, there are several different kinds of biomarkers.

6 The reasoning, here, seems to hinge on the assumptions that there will be if not a cure available for AD, so at least treatment that will modify the disease in a positive way (such as temporarily stopping the progression), and that this treatment is more effective if started in a preclinical stage of AD.

7 The relation between disease and illness in Boorse’s approach is also noteworthy. For Boorse (1975, p. 56), illness refer to “a subclass of diseases, namely, those diseases that have certain normative features reflected in the institutions of medical practice;” an illness must be “a reasonably serious disease with incapacitating effects that make it undesirable”.

8 A normativist critique of Boorse's theory is that specifying a reference class, as is done in his conception, necessarily involves values judgements about the limits of the class. I thank one of the reviewers for emphasising this.

9 Dubois et al. (2016, p. 1121) aptly describe the question of whether the diagnosis of AD should require “the expression of the clinical symptoms such as the first cognitive changes” as a “particular point of contention.”

10 Please note that to say that the diagnosis of AD is “uncoupled from a particular threshold of severity” (Dubois et al. 2010, p. 1120) does not mean that severity does not matter at all in discussions of AD understood as a continuum. See, for example, the IWG’s discussions of mild cognitive impairment (MCI) and AD dementia (Dubois et al. 2014) and between MCI and AD (Dubois et al. 2016), for discussions of distinctions/boundaries and the topic of severity. For example, Dubois et al. state (2014, p. 615) that the IWG criteria from 2007 “enabled AD diagnosis to be extended into the prodromal stage, where the disease can be diagnosed with supportive biomarkers. The ambiguous nature of any categorical boundary between prodromal AD and AD dementia argues in favour of a unified and dimensional diagnostic approach that uses the same criteria for diagnosis irrespective of the severity of cognitive and functional deficits. The recognition of prodromal AD was an important advance over the broader and more heterogeneous state of mild cognitive impairment (MCI).” Discussions of MCI are also present in more recent texts, such as Dubois 2018 (p. 1062), where MCI is described as a “diagnostic label [that] is applied if there is no disease to which MCI can be attributed”, while AD “refers to the whole spectrum of the clinical phase of the disease and is not restricted to the dementia syndrome.” My point, here, is this: this differentiation should be acknowledged, while also acknowledging that the IWG emphasises that symptoms do not need to reach a *specific severity* for a diagnosis of AD to be made.

11 A Hacking-framed concern is related to the one expressed by Schermer and Richard, who cautioned against terms such as “preclinical AD” if used in the clinic. They explain that giving “someone a diagnosis can be understood as a ‘speech act’: it turns a healthy person or research participant into a patient, which has considerable psychological and social consequences and may be harmful” (Schermer and Richard 2019, p. 143). However, the concern with the looping effect is specifically that people may be categorised in new ways, take up these themselves, that such a categorisation/taking up may have looping effects in terms of, for example, strengthening certain understandings of AD and calls for AD biomarker tests, and that the meaning of preclinical AD may be modified in the process.

12 Different conceptualisations also affect *which* people are diagnosed with AD. On the one hand, since not everyone who is identified as having AD biomarkers will develop AD symptoms, the number of people diagnosed with AD can be larger with the NIA-AA conceptualisation than it is with IWG conceptualisation. The latter, as has been shown, requires that symptoms be present. On the other hand, not everyone with dementia symptoms has AD biomarkers, if current biomedical research is correct (Jack et al. 2018). *Partly different people* will be diagnosed with AD, depending on how AD is understood, and *the number of people* diagnosed may depend on the conceptualisation used.
